# Molecular Basis of Resistance to Fusarium Ear Rot in Maize

**DOI:** 10.3389/fpls.2017.01774

**Published:** 2017-10-12

**Authors:** Alessandra Lanubile, Valentina Maschietto, Virginia M. Borrelli, Lorenzo Stagnati, Antonio F. Logrieco, Adriano Marocco

**Affiliations:** ^1^Department of Sustainable Crop Production, Università Cattolica del Sacro Cuore, Piacenza, Italy; ^2^Institute of Sciences of Food Production, National Research Council, Bari, Italy

**Keywords:** *Fusarium*, ear rot, fumonisins, genetic resistance, *Zea mays*

## Abstract

The impact of climate change has been identified as an emerging issue for food security and safety, and the increased incidence of mycotoxin contamination in maize over the last two decades is considered a potential emerging hazard. Disease control by chemical and agronomic approaches is often ineffective and increases the cost of production; for this reason the exploitation of genetic resistance is the most sustainable method for reducing contamination. The review focuses on the significant advances that have been made in the development of transcriptomic, genetic and genomic information for maize, *Fusarium verticillioides* molds, and their interactions, over recent years. Findings from transcriptomic studies have been used to outline a specific model for the intracellular signaling cascade occurring in maize cells against *F. verticillioides* infection. Several recognition receptors, such as receptor-like kinases and *R* genes, are involved in pathogen perception, and trigger down-stream signaling networks mediated by mitogen-associated protein kinases. These signals could be orchestrated primarily by hormones, including salicylic acid, auxin, abscisic acid, ethylene, and jasmonic acid, in association with calcium signaling, targeting multiple transcription factors that in turn promote the down-stream activation of defensive response genes, such as those related to detoxification processes, phenylpropanoid, and oxylipin metabolic pathways. At the genetic and genomic levels, several quantitative trait loci (QTL) and single-nucleotide polymorphism markers for resistance to Fusarium ear rot deriving from QTL mapping and genome-wide association studies are described, indicating the complexity of this polygenic trait. All these findings will contribute to identifying candidate genes for resistance and to applying genomic technologies for selecting resistant maize genotypes and speeding up a strategy of breeding to contrast disease, through plants resistant to mycotoxin-producing pathogens.

## Introduction

A large number of fungi can attack and invade developing maize ears and kernels, causing numerous diseases classified as ear rots. Many ear rot fungi produce mycotoxins that can affect the quality and marketability of grains. *Fusarium verticillioides* (Sacc.) Nirenberg (synonym *F. moniliforme* Sheldon, teleomorph *Gibberella moniliformis* Wineland) causes stalk rot and ear rot in maize, and is endemic in maize fields at harvest (Bottalico, 1998; Battilani et al., 2008). *F. verticillioides* is the main causal agent of Fusarium ear rot (FER) (Logrieco et al., 2002; Folcher et al., 2009). Interest in *F. verticillioides* has been renewed by the discovery that the fungus can produce the secondary metabolite fumonisins (Gelderblom et al., 1988).

Breeding for resistance to FER and fumonisin contamination is considered the environmentally safest and most economical strategy (Munkvold, 2003a; Eller et al., 2008a), and many studies have focused on the search for resistance (Clements et al., 2004; Lanubile et al., 2011; Maschietto et al., 2017). These studies have demonstrated genetic variation for resistance to FER and fumonisin contamination, but no evidence of complete resistance to the pathogen has been observed. Quantitative trait loci (QTL) mapping studies in maize have indicated that resistance is a quantitative trait determined by polygenes having small effect (Pérez-Brito et al., 2001; Robertson-Hoyt et al., 2006; Ding et al., 2008; Chen et al., 2012; Maschietto et al., 2017). Large genetic bases and the strong influence of the environment have slowed progress in accurate QTL localization, therefore reducing the efficiency of marker-assisted selection (MAS) (Robertson-Hoyt et al., 2006). Increasing population size and the number of markers used, improving ear rot phenotyping protocols and integrating data from multiple environments, will overcome such limitations (Robertson et al., 2005).

Transcriptomic and genome-wide association studies (GWAS) are useful tools for identifying candidate genes, especially when combined with QTL mapping in order to map and validate loci for quantitative traits (Korte and Farlow, 2013). The combination of these methods has overcome the limitations of either method performed alone (Brachi et al., 2010). Two recent GWASs were performed in maize to detect SNP associated with increased resistance to FER, resulting in 10 SNP markers with significant effects on several chromosomes (Zila et al., 2013, 2014).

As an alternative to plant breeding techniques, next-generation precision genome engineering relying on genome editing technologies can play a key role in accessing genetic resources and using them to increase plant disease resistance, by targeting suitable plant defense mechanisms. Such approaches, however, require efficient transformation protocols as well as extensive genomic resources and accurate knowledge, before they can be efficiently exploited in practical breeding programs.

In this review, we provide an extensive overview of recent developments related to basic research and breeding efforts aimed at improving resistance to FER and fumonisin contamination in one of the most important grain food crops, i.e., maize.

## Importance of Fusarium Ear Rot Disease

*Fusarium verticillioides*, often in association with *F. subglutinans* and *F. proliferatum* (Logrieco et al., 2002), causes FER or pink ear rot, typically occurring on random groups of kernels or on physically injured kernels (White, 1999; Munkvold, 2003a; Lanubile et al., 2014a). FER prevails in drier and warmer climates, like those common in southern Europe and the United States (Logrieco et al., 2002; Eller et al., 2008a). FER strongly affects grain production, with yield reduction often estimated between 10 and 30% (Bottalico, 1998; Logrieco et al., 2002).

The interest in this fungus has arisen from mycotoxin accumulation in pre-harvest infected plants or in stored grains. *F. verticillioides* mycotoxins, including fumonisins, have been associated with chronic or acute mycotoxicoses in livestock. Feeds contaminated with FB1 caused leukoencephalomacia in horses and pulmonary edema and hepatic syndrome in swine (Ross et al., 1990). FB1 carcinogenic activity in rats (Gelderblom et al., 1996) and its relation with neural tube birth defects in humans (Missmer et al., 2006) have led to the classification of FB1 as carcinogenic for animals and humans. It has been estimated that 25% of world food crops are affected by mycotoxins, but for fumonisins the percentage could be even higher (Bottalico, 1998; Logrieco et al., 2002; Pietri et al., 2004; Eller et al., 2008a).

Regulations for permitted mycotoxin limits in food and feed have been set in most countries (Ferrigo et al., 2016). The European Commission has indicated maximum tolerable levels for fumonisins as 4000 ppb in unprocessed maize, 1000 ppb in maize intended for direct human consumption, and 800 ppb in maize-based breakfast cereals and snacks. Outside Europe, in the main maize producing countries, the US Food and Drug Administration (FDA) has recommended that fumonisin levels in dry milled corn products and cleaned maize used for popcorn should not exceed 2000 and 3000 ppb, respectively. The Health Surveillance Agency for Brazil (ANVISA) has established maximum tolerable limits of 1500 and 1000 ppb in maize meal and other maize-based products, respectively. Furthermore, the permissible levels of fumonisins in maize flour are not more than 200 ppb for the Russian Federal Service for Surveillance on Consumer Rights Protection and Human Wellbeing (Rospotrebnadzor). The different regulations on mycotoxin levels are due to a global market, and since European regulations appear stringent, a common strategy would seem to be the best way forward to ensure food safety.

## *Fusarium verticillioides* Infection in Maize Kernels

**FIGURE 1:**
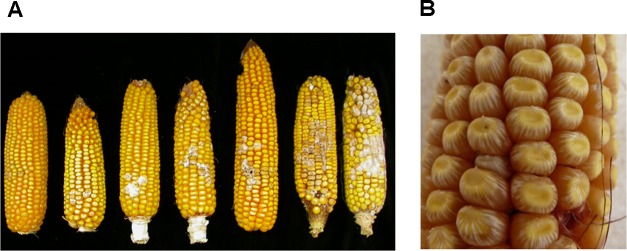
Fusarium ear rot (FER) symptoms. **(A)** Different degrees of FER on ears of resistant (right) to highly susceptible maize lines (left). **(B)** Starburst showing white streaks radiating from the point of silk attachment at the cap of the kernel or from the base. Panel **(A)** is adapted from Maschietto et al. (2017).

*Fusarium verticillioides* has been shown to behave as an endophytic fungus that tends to be symptomless in the kernels and can be systematic in the maize plant (Munkvold et al., 1997). Whitish pink fungal growth on kernels and/or silks is typical. Infected kernels may also exhibit a “starburst” symptom, i.e., white streaks radiating from the point of the silk attachment at the cap or from the base of the kernel (**Figure 1**). There are three main access pathways for the fungus into the ear: (i) fungal spores germinating on the silks and then fungal mycelia growing down the silks to infect the kernels and the cob (rachis); (ii) through wounds on the ear generated by insects, birds, or hail damage; (iii) systemic infection of the ear through infected stalks that generate infected seeds (Munkvold et al., 1997; Munkvold, 2003a). Kernel infection develops most efficiently from strains that are inoculated into the silks (Munkvold et al., 1997), but the prevalence of one or the other pathway depends on the insect pressure in the area.

Only recently the biology of maize kernel infection was investigated using a fluorescent-protein expressing transformant of *F. verticillioides* (Duncan and Howard, 2010). After the introduction of a conidial suspension through the silk channel, the fungus penetrated kernels via the stylar canal and spread within the pericarp, colonizing adjacent cells through pits. Starburst symptoms were observed only at the later times of inoculation, indicating the destruction of the pericarp cell wall (Duncan and Howard, 2010). Early reports focused on germinating seeds revealed that *F. verticillioides* penetrated directly by hyphae through the epidermal cells of the seedling and colonized the host tissue by inter- and intracellular modes of growth (Murillo et al., 1999; Oren et al., 2003). Scutellum colonization occurred earlier with branched hyphae growing into the parenchyma cells, and produced pronounced cell alterations and collapsed protoplasts. Pathogen ingress into the infected tissue induced defense-related ultrastructural modifications, such as appositions on the outer host cell wall surface, the occlusion of intercellular spaces, and the formation of papillae. Pathogenesis-related proteins from maize (PRms) represent the first barrier for fungal penetration and accumulated at very high levels in the aleurone layer and scutellar epithelial cells, as well as within the papillae. This suggests that signaling mechanisms that lead to their accumulation can operate at a distance from the infection point (Murillo et al., 1999).

## Maize–*Fusarium verticillioides* Molecular Interaction

Next-generation sequencing (NGS) and microarray approaches have been used to identify molecular mechanisms connected with *F. verticillioides* infection in resistant and susceptible maize genotypes (Lanubile et al., 2010, 2012a, 2014b; Campos-Bermudez et al., 2013; Wang et al., 2016). All these studies compared the response of resistant and susceptible lines to infection, considering early [12–48 h post-inoculation (hpi)] and late (from 72 to 120 hpi) stages of infection. Microarray hybridization studies were performed in the earliest published works (Lanubile et al., 2010, 2012a; Campos-Bermudez et al., 2013), whereas RNASeq technology has been employed in the more recent references (Lanubile et al., 2014b; Wang et al., 2016). Most of the information about differentially expressed genes has been obtained from infected maize kernels (Lanubile et al., 2010, 2012a, 2014b; Wang et al., 2016), whereas only two experiments have focused on infected silks (Lanubile et al., 2010; Campos-Bermudez et al., 2013). RNASeq has allowed for the identification of several thousands of differentially expressed genes and led to the possibility of detecting new expressed genes (Lanubile et al., 2014b; Wang et al., 2016).

A specific model for the intracellular signaling cascade against *F. verticillioides* infection occurring in maize cells is proposed by the integration of transcriptomic results deriving from Campos-Bermudez et al. (2013), Lanubile et al. (2014b), and Wang et al. (2016).

The first line of defense in plants is the recognition of conserved molecules characteristic of many microbes. These elicitors are also known as microbe-associated molecular patterns (MAMPs). Fungal enzymes breaching the plant cell wall produce oligogalacturonides that are typical MAMPs and elicit defense responses (Ridley et al., 2001; Sanabria et al., 2008; Boller and Felix, 2009). In maize the well-characterized β-1,3-glucanases and chitinases (Lanubile et al., 2012a) may be involved in the degradation of cell walls of *F. verticillioides*, releasing MAMPs-derived cell wall fragments.

Recognition of MAMPs by pattern recognition receptors (PRRs) that are plasma membrane localized receptor-like kinases (RLKs) or receptor-like proteins (RLPs; Boutrot and Zipfel, 2017; Zhang et al., 2017) triggers MAMP-triggered immunity (MTI), thereby reinforcing the host defenses. Several PRRs, including cysteine-rich receptor-like kinase (CRRK), leucine-rich receptor-like kinase (LRRK), RLK, serine threonine kinase (STK), and BRASSINOSTEROID INSENSITIVE 1-associated receptor kinase 1 (BAK1) were identified in transcriptomic studies (Lanubile et al., 2014b; Wang et al., 2016).

A second line of the plants’ defense is recognition of a given effector through a set of plant resistance (*R*) gene products resulting in effector-triggered immunity (ETI) (Jones and Dangl, 2006; Pel and Pieterse, 2013). *R* genes have been found in the interaction maize–*F. verticillioides* belonging to coiled coil-nucleotide binding site-leucine rich receptors (CC-NBS-LRR), NBS-LRR, and nucleotide-binding adaptors shared by APAF-1, R proteins, and CED-4 (NB-ARC) families (Lanubile et al., 2014b; Wang et al., 2016).

**FIGURE 2:**
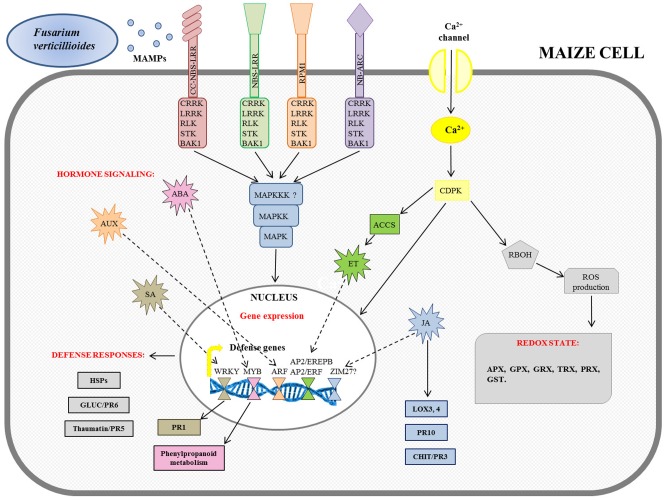
Schematic overview of maize defense gene activation in response to *Fusarium verticillioides* infection. The figure integrates the transcriptomic results previously reported in Campos-Bermudez et al. (2013), Lanubile et al. (2014b), and Wang et al. (2016). MAMPs, microbe-associated molecular patters; NBS-LRR, nucleotide binding site-leucine rich receptor; CC-NBS-LRR, coiled coil-NBS-LRR; NB-ARC, NB-adaptor shared by APAF-1, R proteins, and CED-4; BAK1, BRASSINOSTEROID INSENSITIVE 1-associated receptor kinase 1; CRRK, cysteine-rich receptor-like kinase; LRRK, leucine-rich receptor-like kinase; RLK, receptor-like kinase; STK, serine threonine kinase; CDPK, calcium-dependent protein kinase; MAPK, mitogen-activated protein kinase; MAPKK, MAPK kinase; MAPKKK, MAPKK kinase; RBOH, respiratory burst oxidase homolog protein; ROS, reactive oxygen species; APX, ascorbate peroxidase; GPX, glutathione peroxidase; GRX, glutaredoxin; TRX, thioredoxin; PRX, peroxidase; GST, glutathione-*S*-transferase; ACCS, ACC synthase; ET, ethylene; AP2/EREPB, APETALA2/ethylene-responsive element binding protein; AP2/ERF, AP2/ethylene responsive factor; AUX, auxin; ARF, auxin response factor; ABA, abscisic acid; SA, salicylic acid; PR1, pathogenesis-related 1; HSPs, heat shock proteins; CHIT, chitinase; GLUC, glucanase; JA, jasmonic acid; LOX, lipoxygenase.

Both MTI and ETI triggered down-stream signaling networks in coordination with mitogen-associated protein kinase (MAPK) cascades, as reported in **Figure 2**.

In parallel, Ca^2+^ signaling through the cell membrane could be due to the induction of a specific calcium-dependent protein kinase (CDPK) gene expression after infection (Lanubile et al., 2014b). In turn, several CDPKs also activated respiratory burst oxidase homolog (RBOH) protein to induce early ROS production. The rapidly produced ROS affected the cellular oxidation state, inducing ascorbate peroxidase (APX), glutathione peroxidase (GPX), glutaredoxin (GRX), thioredoxin (TRX), peroxidase (PRX), and glutathione-*S*-transferase (GST) gene expression, involved in plant cell wall reinforcement (Campo et al., 2004; Mohammadi et al., 2011). It has been shown that in resistant maize seedlings, before infection, APX and superoxide dismutase (SOD) enzymatic activities were higher than in the susceptible ones, while 5 days after inoculum, they remained unchanged. On the other hand, in the susceptible seedlings all enzymes assayed were activated only after *F. verticillioides* infection (Lanubile et al., 2012b).

These signals are primarily orchestrated by hormones until they reach the nucleus (Berens et al., 2017). The involvement of hormone-signaling genes, including salicylic acid (SA), auxin (AUX), abscisic acid (ABA), ethylene (ET), and jasmonic acid (JA), has been observed (**Figure 2**). The targets of the hormone-signaling transduction pathways have been found to be multiple transcriptional factor families, such as WRKY for SA, MYB for ABA, auxin response factor (ARF) for AUX, and APETALA2/ethylene-responsive element binding protein (AP2/EREPB) and AP2/ethylene responsive factor (AP2/ERF) through 1-aminocyclopropane-1-carboxylate (ACC) oxidases for ET (Campos-Bermudez et al., 2013; Lanubile et al., 2014b; Wang et al., 2016). WRKY are normally involved in the signal transduction pathway because they recognize the W-box of promoters of a large number of defense-related genes; in particular their association with the *PR1* gene has been described (Campos-Bermudez et al., 2013; Wang et al., 2016). Furthermore, it has been reported that Myb-like DNA binding proteins are involved in the signaling cascade for flavonol-specific gene activation in phenylpropanoid biosynthesis (Lanubile et al., 2014b). Other changes observed after *F. verticillioides* infection comprise the activation of genes encoding heat shock proteins (HSPs) as well as glucanases (GLUC or PR6) and thaumatin or PR5 proteins (Campos-Bermudez et al., 2013; Lanubile et al., 2014b; Wang et al., 2016). A JA signaling pathway has been found to promote the further down-stream activation of defense responsive genes for PR proteins, such as chitinases (CHIT or PR3) and PR10, and lipoxygenases (LOX3; LOX4). The role of JA in maize pathogen defense has recently been reviewed (Borrego and Kolomiets, 2016; Lim et al., 2017), and the relevance of genes for the lipoxygenase pathway in resistance to *F. verticillioides* is well established.

*LOX* genes have been found across animal, fungal, and plant kingdoms, and are presumed to be involved in plant susceptibility to fungal invasion and mycotoxin production (Kock et al., 2003; Christensen and Kolomiets, 2011; Maschietto et al., 2015). *LOX* genes are non-heme iron-containing dioxygenases that catalyze the oxygenation of polyunsaturated fatty acids (PUFAs) (Vick and Zimmerman, 1983), which are processed into an estimated 400 metabolites including the well-known hormone JA and green leaf volatiles (GLVs) (Mosblech et al., 2009). *LOX* genes are subdivided into two main functional groups, 9-LOXs and 13-LOXs, depending on which carbon on the fatty acid chain is oxygenated. A total of 13 different maize *LOXs* (*ZmLOXs*) with varying functions, localization, and regulation within the plant, have been reported (Yan et al., 2012). Of the 13 *ZmLOXs*, *ZmLOX4* and *ZmLOX5* located on chromosome 5 are the two most closely related paralogs, sharing only 40–67% of sequence identity with other *ZmLOXs* (Park et al., 2010). *ZmLOX4* and *ZmLOX5* are 9-LOXs and are segmentally duplicated genes. Other pairs of close paralogs include tandemly duplicated *ZmLOX1* and *ZmLOX2* and segmentally duplicated genes *ZmLOX7* and *ZmLOX8*, and *ZmLOX10* and *ZmLOX11* (Nemchenko et al., 2006; Christensen et al., 2013).

Maize mutants for a defective 9-LOX gene, *ZmLOX3*, resulted in reduced levels of several 9-LOX-derived fatty acid hydroperoxides. *F. verticillioides* conidiation and FB1 production, as well as other fungal diseases, were drastically reduced in kernels of *lox3* mutants (Gao et al., 2007, 2009). In addition, maize 9-LOX *ZmLOX12* suppressed contamination by *F. verticillioides* (Christensen et al., 2014). These observations suggest that a specific plant 9-LOX isoform is required for fungal pathogenesis, including disease development and production of spores and mycotoxins.

Localization and expression data supported the hypothesis that another *LOX* gene, *ZmLOX5* (expressed in the silks), affected resistance to other mycotoxigenic fungi, and a QTL affecting aflatoxin contamination was located where *ZmLOX5* also mapped (Warburton et al., 2010).

Key genes in the defense response are those of the phenylpropanoid pathway, encoding for phenylalanine ammonia lyase and chalcone synthase, leading to an accumulation of flavonoids, phenolic compounds, and phytoalexins. Phenolic compounds accumulate rapidly during host–pathogen interaction and may mediate disease suppression through the inactivation of fungal enzymes or the strengthening of plant structural components. High levels of phenylpropanoids in the kernel pericarp were associated with less severe FER and fumonisin accumulation (Assabgui et al., 1993; Sampietro et al., 2013). The most resistant genotypes exhibited high levels of phenylpropanoids (on average 23.7 mg/g of dry pericarp), related to low levels of disease severity and grain fumonisin concentration (5.6% of visibly diseased ear area and 56.7 ppm of fumonisin on average, respectively; Sampietro et al., 2013). In particular, total diferulates were the best explanatory parameter for the variability of disease severity, and grain fumonisin concentration was correlated to total diferulate, 8,5′-diferulic acid benzofuran, and p-coumaric acid content. A potent inhibitory effect of α-tocopherol (0.1 mM) and ferulic acid (1 mM) on fumonisin biosynthesis was observed in *F. verticillioides* liquid cultures (Picot et al., 2013). These antioxidants were present in all stages of maize kernel development, indicating that the fumonisin-producing fungi were likely to face them during ear colonization.

Flavones in the silks contribute to FER resistance (Reid et al., 1992). Sekhon et al. (2006) investigated silk and kernel resistance to *F. verticillioides* and *F. proliferatum* in maize lines differing in 3-deoxyanthocyanidins and related 3-deoxyflavonoid (flavan-4-ols) content. Even though the degree of resistance was not strictly proportional to the amount of these secondary compounds in silks, the genes of the flavonoid pathway were active during the early stages of silk development. However, upon fungal inoculation, accumulation of 3-deoxyanthocyanidins was observed in resistant lines, suggesting a role of these compounds in resistance to *F. verticillioides*.

Higher susceptibility to FER was shown in ears of the *brown midrib* (*bm3*) mutant of maize, which cannot methylate either caffeic or hydroxyferulic acids to ferulic or sinapic acids due to a mutated *O*-methyltransferase (Vignols et al., 1995). Of the secondary metabolites, 6-methoxybenzoxazolin-2(3H)-one (MBOA) and benzoxazolin-2(3H)-one (BOA) have been found in corn and they are known for their antimicrobial properties (Glenn et al., 2002). Nevertheless, *F. verticillioides* is able to detoxify these compounds thanks to the presence of two specific loci, *Fdb1* and *Fdb2* (Glenn et al., 2002). Benzoxazinones are detoxified in 2-aminophenol (AP), which is converted to the less toxic *N*-(2-hydroxyphenyl) malonamic acid (HPMA) (Bacon et al., 2007). An endophytic bacterium, *Bacillus mojavensis*, is considered efficacious as a control of this *Fusarium* species, because it is able to produce a pigment identified as 2-amino-3H-phenoxazin-3-one (APO), which interacts with the fungus, thus preventing the usual transformation of AP into the non-toxic HPMA. The higher amounts of APO are toxic to *F. verticillioides* (Bacon et al., 2007).

The role of the biochemical composition of the endosperm has also been investigated. In particular, although Snijiders (1994) concluded that the biochemical composition of the endosperm had no intrinsic effect in proteins, sugars, and starches on resistance to the pathogen, Bluhm and Woloshuk (2005) found an influence on fumonisin B1 biosynthesis. Low amounts of amylopectin, required for fumonisin B1 biosynthesis, in early stages of kernel development and in some maize mutants, correlated with lower levels of mycotoxins (Bluhm and Woloshuk, 2005). The dynamic of water activity and humidity of maize kernels and their relevance for fumonisin accumulation in kernels was studied in medium to late season commercial hybrids by Battilani et al. (2011). The study revealed how “slow dry down” hybrids were more prone to fumonisin accumulation, irrespective of their maturity class.

More recently, the effect of fatty acid composition on fumonisin contamination and the occurrence of hidden fumonisins in maize (masking phenomenon consisting in the formation of covalent bonds between the tricarballylic groups of fumonisins and the hydroxyl groups of starch or the amino or sulfhydryl groups of the side chains of amino acids in proteins) has been investigated: higher fumonisin contamination was measured in hybrids showing a higher linoleic acid content and a higher masking action was observed in hybrids with higher oleic to linoleic ratio (Dall’Asta et al., 2012). Unsaturated fatty acids are often oxidized to produce oxylipins, whose role as signal molecules that regulate the response to biotic stress has been previously described (Wilson et al., 2001; Christensen and Kolomiets, 2011).

In general, it is worth mentioning that basal defense mechanisms against *F. verticillioides* were activated in maize-resistant kernels, as reported in several studies. Many proteins associated with the defense response were found to be more abundant after infection, including PR10, chitinases, xylanase inhibitors, proteinase inhibitors, and PRXs. Kernels of the resistant line, even the non-inoculated ones, contained higher level of these defense-related proteins than the susceptible line, suggesting that these proteins may provide a basal defense against *Fusarium* infection in the resistant line (Mohammadi et al., 2011). These findings confirmed the conclusions of Lanubile et al. (2010, 2015a) and Maschietto et al. (2016) based on a transcriptomic analysis of the same resistant lines. Similar results were also obtained by Campos-Bermudez et al. (2013) using transcriptional and metabolite analysis in different resistant and susceptible inbreds. These results indicated that resistance was due to constitutive defense mechanisms preventing fungal infection. These mechanisms were poorly expressed in the susceptible line and, although the inoculation activated the defense response, this was not enough to prevent the disease’s progress.

## Genetic Basis of the Resistance to *Fusarium* Infection

A deeper knowledge of the genetic basis underlying FER is necessary to speed up progress in breeding for resistance.

The most efficient way to improve FER resistance in hybrids is to evaluate and select among inbred lines, before using resources to produce hybrids (Hung and Holland, 2012). Lanubile et al. (2011) conducted screening trials for both FER and fumonisin concentration using public and private inbred lines, and identified several genotypes with good levels of resistance to both FER and fumonisin accumulation. In diallel mating of 18 inbred lines from different heterotic groups with different levels of resistance, hybrids had 27% less ear rot and 30% less fumonisin content than their inbred parents (Hung and Holland, 2012). General combing ability (GCA) and specific combining ability (SCA) were significant for disease resistance, and inbred performance *per se* and the corresponding GCA in hybrids were significantly correlated (*r* ≥ 0.78).

Fusarium ear rot resistance has proved to be a quantitative trait determined by polygenes (Pérez-Brito et al., 2001; Robertson-Hoyt et al., 2006; Eller et al., 2008b). Pérez-Brito et al. (2001) tested two F_2_ tropical maize populations of 238 and 206 F_2_ individuals derived, respectively, from single crosses between resistant and susceptible inbred lines for FER resistance, and they measured relatively low heritability (*h*_2_ = 0.26–0.42). Robertson-Hoyt et al. (2006) tested two segregating populations of 213 BC_1_F_1:2_ families from the first backcross of GE440 to FR1064 (GEFR) and 143 recombinant inbred lines (RILs) from the cross of NC300 to B104 (NCB), respectively, both for fumonisin contamination and FER resistance traits. This experiment enhanced the breeding for resistance approach because family mean heritability for ear rot resistance increased by up to 0.47–0.80 and for fumonisin contamination by up to 0.75–0.86. The increment of the heritability in comparison to Pérez-Brito’s experiment can be explained by a reduction in the environmental influence obtained by doubling the number of evaluation environments and the number of artificial inoculations per plant. High positive correlations of FER resistance with fumonisin contamination and moderate-high heritabilities of both traits observed in the populations GEFR and NCB suggested that selecting for both traits at the same time was feasible (Robertson et al., 2006).

Phenotypic correlation between the severity of FER and the amount of fumonisins has been reported to be moderate to low (Clements et al., 2003; Clements et al., 2004), probably because of symptomless endophytic infections (Oren et al., 2003). Moreover, genotypic correlation between the two traits was higher than the phenotypic correlation (0.87–0.96 versus 0.40–0.64) (Robertson et al., 2006). This demonstrated that genotypic effects on susceptibility to ear rot and fumonisin content were highly correlated (Robertson et al., 2006). The close correlation between FER and fumonisin accumulation suggests that toxin analysis is only rarely needed, if disease severity data are available. In breeding, selection against genotypes more susceptible to FER allows for simultaneous selection against genotypes accumulating high contents of fumonisins. Moreover, genetic mechanisms controlling both traits are the same or closely linked.

## Maize Quantitative Trait Loci (QTL) Providing Resistance to *Fusarium verticillioides*

The response to selection for resistance to FER can be increased by a wide variability in maize genotypes toward disease resistance and fumonisin contamination and by the moderate to high heritability of the traits. Nevertheless, phenotypic selection for the two traits is hampered by practical difficulties. Although many diseases could be evaluated during the plant’s young stage or before flowering, FER and mycotoxin concentrations can only be analyzed on mature seeds and require artificial inoculations with calibrated fungal spore suspensions for consistent evaluation of the disease (Clements et al., 2003). Moreover, asymptomatic infections of this pathogen lead to time-consuming and expensive toxin assays for contamination assessment.

In addition, plant traits can affect pathogen access through the silk channel and the kernel. Hybrids with tight, adherent husks, and less open apical parts of the ear were more resistant to FER (Warfield and Davies, 1996; Butron et al., 2006). Physiological traits such as earliness in flowering time have been shown to reduce susceptibility toward several pathogens, including *F. verticillioides*. FER was less common for inbred lines with green and actively growing silks at inoculation time rather than brown silks. Kernel properties and seed coat influenced pathogen success (Scott and King, 1984; Headrick and Pataky, 1991; Hoenish and Davis, 1994). A thicker pericarp made maize more resistant to penetration. Disease severity was dependent on husk integrity, on drought stress that increased the amount of stalk rot, and agronomic practices, for instance irrigation at the silk stage (Munkvold, 2003b; Battilani et al., 2008).

Finally, FER is influenced by many environmental factors, and testing for multiple sites and years is required (Shelby et al., 1994; Munkvold, 2003a; Robertson et al., 2006; Zila et al., 2013; Maschietto et al., 2017).

### Disease Phenotyping

Selection for resistant hybrids must occur in areas with a known high incidence of FER. *F. verticillioides* can over-winter in the soil and may be spread by wind, rain splash, and insect larvae (Munkvold, 2003a), but to ensure equal distribution of the pathogen for all of the plants in the field, artificial inoculation is needed (Munkvold and Desjardins, 1997). Kernel infection through the seeds and infection through the silks are the best techniques for evaluating genetic resistance to FER (Munkvold and Desjardins, 1997; Robertson et al., 2006). These techniques refer to two types of inoculation method: with (type 1) and without (type 2) mechanical inoculation. Type 1 methods include toothpick inoculation methods and test kernel resistance (Reid et al., 1996), whereas in a typical type 2 method, a spore suspension is sprayed onto the maize silks with an atomizer, or injected into the silk channel near the cob tip. Type 1 inoculation methods usually screen for resistance to spreading on the host and simulate insect attack, as they bypass many of the plant’s morphological barriers. Type 2 inoculation methods more closely resemble natural infection of a non-wounded host plant.

The best differentiation between resistant and susceptible genotypes has been obtained when inoculation occurred within a week after silking for type 2 inoculation (Reid et al., 1992; Lanubile et al., 2010; Campos-Bermudez et al., 2013); type 1 inoculation was effective 15 days after pollination (Lanubile et al., 2014b; Wang et al., 2016). Later inoculations resulted in significantly less severe disease symptoms, while the very early ones, i.e., 4–6 days, increased cases of disease outbreak.

As an alternative to field tests, *in vivo* bioassays including the rolled towel assay (RTA) or the Petri dish bioassay have been proposed for testing the ability of different pathogens to infect and colonize seedlings and kernels, respectively (Ellis et al., 2011; Lanubile et al., 2015b; Ju et al., 2017).

Fungal contamination of grains can be measured by various methods: the ergosterol level, representing a quantitative and qualitative measure of fungal contamination (Bakan et al., 2002), even though it is not strictly correlated with mycotoxin content; and the absolute quantification of fungal housekeeping genes, such as β-*tubulin*, through quantitative PCR (Lanubile et al., 2010, 2012a, 2014b).

Accurate mycotoxin analysis can be conducted with high-performance liquid chromatography (HPLC), but its costs make this technique unsuitable for use in large-scale breeding programs. HPLC can be replaced by the ELISA assay (Eller et al., 2008a) and near-infrared spectroscopy (NIRS) (Siesler et al., 2002; Berardo et al., 2005). The NIRS methodology can potentially be used for large-scale selection of genotypes resistant to fungal and fumonisin contamination.

### Mapping QTL for Resistance and Genome-Wide Association Studies

**FIGURE 3:**
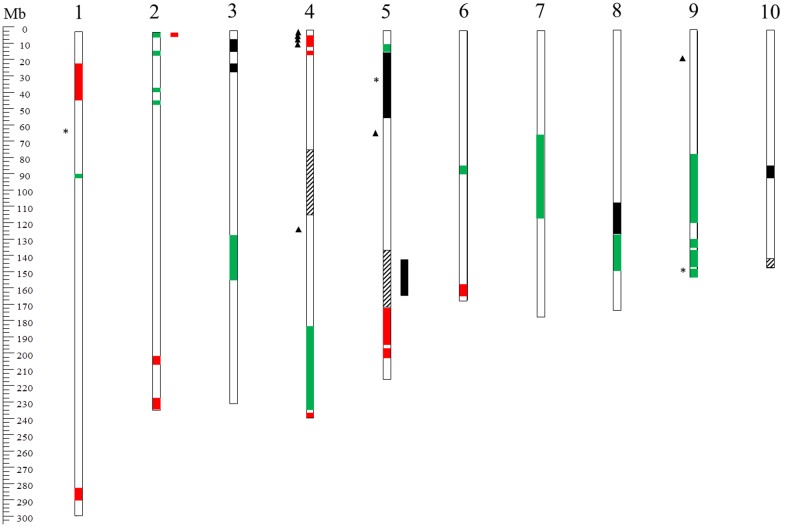
Overview of chromosomal locations on the B73 reference genome (version 2) of known QTL for FER resistance. The bars inside the chromosomes indicate QTL intervals detected by Robertson-Hoyt et al. (2006) (red), Ding et al. (2008) (black), Chen et al. (2012) (dashed), and Maschietto et al. (2017) (green). The asterisks and the triangles on the left side of the chromosomes indicate FER-associated SNPs detected by Zila et al. (2013, 2014), respectively.

Quantitative trait loci mapping and MAS, using PCR-based DNA markers associated to resistance genes, could be a successful strategy for selecting lines resistant to *F. verticillioides* (Beavis, 1998; Robertson et al., 2005). Localization of FER resistance QTL has often appeared to be contradictory in different studies (Pérez-Brito et al., 2001; Robertson-Hoyt et al., 2006; Ding et al., 2008), probably because of a strong environmental influence on the spread of the disease. **Figure 3** reports the localization of the main QTL and SNP markers for FER resistance on the maize chromosomes. Pérez-Brito et al. (2001) identified nine and seven QTL in two F_2_ populations cross 3 × 18 and 5 × 18, respectively. The detected QTL explained between 30 and 44% of the phenotypic variation in the first population and 11–26% in the second. Three QTL on chromosomes 3 and 6 were co-located in both populations. Due to the number and limited effects of the QTL detected, Pérez-Brito et al. (2001) excluded MAS as a suitable strategy for this trait. Further studies contrasted with Pérez-Brito’s conclusion (Robertson-Hoyt et al., 2006; Ding et al., 2008). Robertson-Hoyt et al. (2006) tested two segregating populations, a GEFR and an NCB population, derived, respectively, from FER resistant line GE440 crossed with FR1064 and the low fumonisin contamination line NC300 crossed with B104. In the GEFR population, seven QTL were identified, explaining 47% of the phenotypic variation for FER resistance, and nine were found for fumonisin content, explaining 67% of the variation. In the NCB population, five QTL explained 31% of the FER variation and six QTL explained 81% of the fumonisin variation. Three QTL for FER and two for fumonisin were mapped in similar positions in the two populations. In particular, two QTL, localized on chromosomes 4 and 5, appeared to be consistent for both traits in both populations. Ding et al. (2008) tested a RIL population of 187 genotypes for FER resistance. Of five identified QTL, two on chromosome 3 were stable across environments. The major QTL explained 13–22% of the phenotypic variation for FER, and it was flanked by SSR markers umc1025 and umc1742. More recently, a QTL on chromosome 4 (bin 4.05/06) was identified in the resistant inbred line BT-1 which explained 17.95% of the phenotypic variation for resistance to FER (Chen et al., 2012). Further verification of the QTL effect in near-isogenic lines (NILs) carrying the QTL region on chromosome 4 showed that if homozygous, this QTL can increase the resistance by 33.7–35.2%. The stable and significant resistance effect of the QTL on chromosomes 3 and 4 lays the foundation for further MAS and map-based cloning.

In conclusion, since QTL mapping used populations originated by crossing two homozygous lines, genetic variation in FER resistance was limited to the differences between the two parents. Furthermore, the resolution power was often low and QTL positions spanned from a few to tens of centimorgans. These regions corresponded to several megabases which contained hundreds of genes.

Such limitations, as well as the strong influence of environmental factors, hinder accurate QTL localization and the possibility of performing MAS efficiently (Robertson-Hoyt et al., 2006).

These issues may be partially overcome by increasing population size and the number of markers used, improving ear rot phenotyping protocols and integrating data from multiple environments (Robertson et al., 2005). In particular, initial QTL mapping studies on these traits were based on maps containing a few hundred restriction fragment length polymorphisms (RFLP; Pérez-Brito et al., 2001) and single sequence repeat (SSR) markers (Robertson-Hoyt et al., 2006; Ding et al., 2008; Chen et al., 2012). In recent years, single-nucleotide polymorphisms (SNPs) have become the preferred genotyping system for genetic studies, being the cheapest and the most abundant markers in a genome (Rafalski, 2002), e.g., 1 SNP/100 bp in maize (Tenaillon et al., 2001).

With the advent of NGS technologies, SNP markers have shown their full potential with novel approaches combing SNP discovery and genotyping, such as Genotyping-by-Sequencing (GBS; Elshire et al., 2011).

Three GBS studies were performed on maize to detect allele variants associated with increased resistance to FER. In a maize core diversity panel of 267 inbred lines, three SNPs with significant effects on chromosomes 1, 5, and 9 were described (Zila et al., 2013). Seven SNPs in six genes associated with FER resistance were identified on chromosomes 4, 5, and 9 in a panel of 1,687 US maize inbred line collections (Zila et al., 2014). Maschietto et al. (2017) found eight QTL located on linkage groups (LGs) 1, 2, 3, 6, 7, and 9 that were common to FER response and FB1 contamination, making the selection of genotypes with both low disease severity and low fumonisin contamination possible. Five QTL were located close to previously reported QTL for resistance to other mycotoxigenic fungi. Moreover, combining previous transcriptomic data (Lanubile et al., 2014b) with QTL mapping, 24 candidate genes for resistance to *F. verticillioides* were positioned in the same chromosomal regions.

Furthermore, comparing studies addressed to detection of QTL for resistance against different diseases reveals that there is evidently an overlap of the genetic mechanisms involved. Several fumonisin contamination QTL (Robertson-Hoyt et al., 2006) were localized on chromosomes 1, 2, 3, 4, 5, and 9 close to QTL for aflatoxin contamination (Wisser et al., 2006). In addition, Robertson-Hoyt et al. (2007) discovered QTL affecting both fumonisin and aflatoxin contamination, and Fusarium and Aspergillus ear rots.

## The Role of Fumonisins in the Host–Pathogen Interaction

*Fusarium verticillioides* produces fumonisins as secondary metabolites (Gelderblom et al., 1988), a family of mycotoxins that affects animal and human health (Munkvold and Desjardins, 1997). Among the most active fumonisins, *F. verticillioides* produces B series fumonisins, particularly FB1.

FB1 is synthesized via a polyketide biosynthetic pathway (Butchko et al., 2006). The fumonisin (*FUM*) gene cluster, including genes involved in FB1 biosynthesis, is known to contain 22 genes with a length of 42 kb (Proctor et al., 2003). Of the 22 genes, 15 genes are co-regulated, including the key gene *FUM1*, which encodes a polyketide synthase (PKS) (Proctor et al., 1999).

There are contrasting reports on the role of fumonisin production in the ability of *F. verticillioides* to cause maize ear rot. Fumonisin-nonproducing mutants were generated by disrupting *FUM1*, the gene encoding PKS, which is required for fumonisin biosynthesis (Proctor et al., 1999). *Fum1* mutants were 100% reduced in fumonisin production, but in field tests they were able to cause ear rot. The results provided evidence that production of fumonisins was not required for ear rot development and suggest that it is unlikely that fumonisin resistance would be an effective way to control this disease or fumonisin contamination in maize (Jardine and Leslie, 1999; Desjardins et al., 2002). Conversely, Lanubile et al. (2013) observed an enhanced reaction of incompatibility between resistant host and a *fum1* mutant of *F. verticillioides*, impaired in PKS activity, compared with the isogenic wild-type strain. In the early stages of infection, when the production of fumonisins was not detectable, the *fum1* mutant differed in its ability to colonize maize kernels compared to the wild-type strain. In the resistant maize genotype, the *fum1* mutant provoked a delayed and weakened activation of defense-related genes, presumably as a consequence of reduced growth. The inability of the *fum1* mutant to infect maize ears may be related to PKS activity and its association with the LOX pathway. Plant and fungal *LOX* genes were up-regulated after *fum1* mutant inoculation, suggesting that *PKS* is a relevant gene, essential not only to the fumonisin biosynthetic pathway, but also to pathogen colonization.

Arias et al. (2012) focused on the role of fumonisins as possible pathogenicity factors in the maize–*F. verticillioides* interaction. The effect of fumonisin on the development of maize seedling disease was observed to be strongly influenced by toxin concentration. High levels of fumonisin triggered necrosis and wilting in maize seedlings, while on the other hand low doses activated detoxification processes, suggesting a strategy of recovery in the host plants.

Death induced by FB1 usually presents features which resemble those of the hypersensitive response (HR), being fast and limited to the tissues that are exposed to the toxin (Asai et al., 2000; Stone et al., 2000), and determining the induction of defense genes (pathogenesis-related, phenylalanine ammonia lyase), chromatin condensation, and production of ROS, possibly in the apoplast through peroxydases. Different tissues and species have been used in the past for these toxicity studies, ranging from roots to leaves, from maize to *Arabidopsis* (Stone et al., 2000; Nadubinska and Ciamporova, 2001; Lin et al., 2008; Sánchez-Rangel et al., 2012).

FB1 acts through several pathways: SA, ET, and jasmonates (Asai et al., 2000). It causes a depletion of extracellular ATP reservoirs and eventually involves the protease vacuolar-processing-enzyme (VPE) as regulator of programmed cell death (PCD) (Kuroyanagi et al., 2005). Finally, there is evidence that ubiquitination also plays an important role in FB1-induced PCD (Lin et al., 2008). Future knowledge of the toxicity mechanisms of this molecule might suggest new management strategies.

## Future Prospects

Several omics aspects of the *F. verticillioides*–maize interaction have been discussed in this review. Although down-stream processes of response to *F. verticillioides* infection have been well elucidated through transcriptomic studies, less information is available on the up-stream processes of recognition between maize and the fungus. To fill these gaps, recent advances in genomic technologies, such as GWAS, could resolve this complex trait down to the sequence level (Zhu et al., 2008). Moreover, GWAS applied to a large multi-parent population of RILs, termed multi-parent advanced generation inter-cross (MAGIC; Cavanagh et al., 2008), will ensure the identification of multiple genes, determining resistance to both FER and fumonisin contamination. In addition, as resistance to *F. verticillioides* is quantitative and based on a diffused architecture of many minor genes, the best approach for future molecular breeding will shift from MAS to genomic selection. Genomic-assisted breeding for quantitative resistance will necessitate whole-genome marker profiles for the entire set of breeding lines, prediction models and selection methodology as implemented for classical complex traits such as yield (Poland and Rutkoski, 2017).

A critical issue is that of the exploitation of candidate genes for resistance. RNASeq has been of great value in improving, validating, and refining gene models, and can identify new genes not previously annotated. A new approach to identifying candidate genes and QTL for resistance is represented by plant metabolome investigation after pathogen infection. Growing efforts are being made in research into relating genomic to metabolic (phenotypic) information (Bueschl et al., 2014). Keurentjes et al. (2006) have shown the potential of untargeted metabolomics to reveal QTL in the model plant *Arabidopsis*. An increasing number of metabolites are assigned to specific metabolic pathways and are the products of enzymatic reactions that depend on genome regulation. Moreover, the metabolic profile corresponds to the biochemical status of the organism that is a phenotypic expression. Metabolic profiling of resistant and susceptible cultivars can be used to detect biomarkers associated with the resistant trait.

In addition, genetic engineering permits the introduction or modification of gene coding for proteins with antifungal activities and enzymes that breach the plant cell wall, to increment pathogen resistance. In maize, several transgenic approaches can be exploited to reduce fumonisin content: reducing disease severity either by eliminating insect injury or by decreasing pathogen efficacy, by detoxifying or by blocking the synthesis of mycotoxins in seed (Duvick, 2001; Gao et al., 2007; Yuan et al., 2007). A limitation of this strategy is the possibility that other biosynthetic pathways might be altered, resulting in the biosynthesis of new plant secondary metabolites. Moreover, new identified dominant resistance genes (*R* genes) could be engineered in order to increase resistance in a specific response.

More recently, efficient editing technologies for genome modification in multiple plant species have emerged. Of these, the clustered regularly interspaced short palindromic repeat (CRISPR)-Cas9 system has been used successfully in staple crops to modify single genes and change expression patterns. New gene variants conferred beneficial traits for plant breeding, including stress tolerance (Svitashev et al., 2015; Char et al., 2017; Shi et al., 2017).

Finally, it is now generally accepted that efforts devoted to the improvement of resistance to FER will also determine increases in resistance to other ear rots and, in particular, to the rotting produced by *Aspergillus* spp. Several studies have dealt with the positive relationship between infection by *Fusarium* and *Aspergillus* spp. (Clements and White, 2004; Lanubile et al., 2011; Pechanova and Pechan, 2015). Such results suggest that these fungal species may require similar substances for growth and development, and that they interact in similar ways with the host plant.

## Author Contributions

AL contributed for writing and editing the major part of the review and was involved in approving the final version of the review. VM, VB, and LS organized and prepared some of the parts of this review. AFL critically revised the manuscript. AM contributed to the design of this work outlay and was responsible for drafting the manuscript and final approval from all others.

## Conflict of Interest Statement

The authors declare that the research was conducted in the absence of any commercial or financial relationships that could be construed as a potential conflict of interest.
